# Developing and validating machine learning models to predict acetabular cup size in total hip arthroplasty

**DOI:** 10.1016/j.jor.2025.07.021

**Published:** 2025-07-23

**Authors:** Felix C. Oettl, Aaron I. Weinblatt, Brian Chalmers, David Kolin, Alejandro Gonzalez Della Valle

**Affiliations:** a Hospital for Special Surgery, New York, NY, USA; b Department of Orthopedic Surgery, Balgrist University Hospital, University of Zürich, Zurich, Switzerland

**Keywords:** Total hip arthroplasty, Cup size, Artificial intelligence, Resource utilization

## Abstract

**Aims:**

Adequate implant inventory management can improve efficiency, storage space, and result in cost savings in arthroplasty. This study investigates if the prediction of cup size in elective primary total hip arthroplasty (THA) cound be improved with the use of advanced machine learning.

**Methods:**

Using the arthroplasty registry of a single institution, we identified 30,583 patients who underwent primary THA between 2016 and 2024. No data was missing or incomplete. A total of 9 parameters readily available preoperatively were included as potential predictor variables. The data corpus was partitioned into training (80 %) and hold-out test (20 %) samples. Two distinct machine learning models were trained on regression tasks. The models were technically evaluated utilizing Root Mean Squared Error (RMSE) and Mean Absolute Error (MAE). Spearman correlation coefficient was calculated to assess alignment with implanted cup. 95 % confidence intervals (95 % CI) were calculated via bootstrapping. Real world useability was assessed by the percent of correct predictions within ±2 mm from implanted cup.

**Results:**

The quantile regression forest outperformed the explainable boosted machine (EBM) in terms of MAE (1.69 [95 % CI 1.64, 1.73] vs 1.73 [1.69, 1.77]) and real-world usability, with an accuracy of 82.85 % within ±2 mm and 97.27 % within ±4 mm. The EBM outperformed the QRF by RMSE and Spearman Correlation coefficient, weighing outliers heavier. The most important factors in order were Sex, height, age, weight, surgical approach and BMI.

**Conclusion:**

Machine learning models can predict implant sizing with very high accuracy based on a few metrics available preoperatively. This model can help decrease overall cost of THA by improving orthopaedic manufacturers' supply chains and hospitals’ inventory management.

## 1 Introduction

The demand for total hip arthroplasty (THA) is expected to keep growing, highlighting the increasing importance of efficient resource management in orthopedic surgery.^1^^,^^2^ A key aspect of this efficiency is the ability to accurately predict implant sizes, which can lead to optimized stocking, improved operating room efficiency, and decreased associated costs.^1^^,^^3^

Chen et al. demonstrated a strong association between patient demographics (height, weight, and sex) and implant size, achieving 40.3 % accuracy at ±2 mm and 73.5 % ±4 mm accuracy using Bayesian modeling.^4^ This approach has value for centers without the ability to calibrate radiographs for preoperative templating and for surgeons who determine implant size on the day of surgery. However, with the prior model's performance, it is not ideal for widespread implementation.

Recent efforts have focused on improving the accuracy of preoperative planning by incorporating patient-specific demographics. Pourmoghaddam et al. implemented a predictive model using a multiple regression model which not only included demorgraphics, but also planned implant type. They were able to achieve 93 % accuracy for acetabular component size within ±2 mm.^5^ However, a reliable method to determine required implant sizes based on simple patient demographics alone for THA is still lacking.

The purpose of this study is to utilize and compare advanced machine learning techniques, specifically Quantile Regression Forests (QRF) and Explainable Boosting Machine (EBM) models, to improve acetabular cup size prediction and determine the predictive capacity of specific patient demographics with cup size in THA. We hypothesize that these models, trained on a large dataset, will provide improved predictive modeling of implant sizes compared to previously-published methods.

## 2 Material and methods

### 2.1 Design

This study was conducted at a single academic center. The study adhered to the Transparent Reporting of a Multivariable Prediction Model for Individual Prognosis or Diagnosis (TRIPOD) guidelines as well as the Guidelines for Developing and Reporting Machine Learning Models in Biomedical Research.^6^^,^^7^ The collection of clinical data was undertaken with the approval of the Institutional Review Board.

The study was performed using the institutional arthroplasty registry and comprised of all 30,587 patients undergoing primary THA between March 2016 and January 2024.

The primary outcome was acetabular cup size, as a continuous outcome in millimeters (mm). The secondary outcome was the proportion of correct predictions within ± 2 mm intervals of the implanted cup.

### 2.2 Source data and input

All prediction models were developed using 9 variables – height, sex, age, weight, BMI, surgical approach, computer navigation, robotic assist, and race – all of which are demographic or planned pre-procedure and therefore available before the patient is admitted. For the models unable to receive categorical variables as input, variables were one hot encoded before training the respective models. The analysis of variable importances showed the extent to which each variable affected the model's ability to make predictions. No data was missing or incomplete. The dataset was randomly split into training (80 %) and testing (20 %) sets, with a set seed of 42 to ensure reproducibility.

### 2.3 Model development

Categorical variables were summarized by presenting their absolute frequencies and corresponding percentages, whereas continuous variables were summarized by presenting their means and standard deviations (Table 1). The predictive modeling for the continuous model was performed by employing 2 distinct machine learning algorithms: EBM^8^ and QRF.^9^^,^^10^ EBM is an ensemble method based on decision trees, gaining popularity in recent years due to high accuracy on complex datasets. QRF is a non-parametric ensemble methodology that utilizes tree-based models to estimate conditional quantiles^9^ The 2 models were derived using the training cohort and validated on the test cohort.

**Table 1:** Characteristics of the Training and Testing Sets and complete cohort.

Characteristic	Complete cohort (N = 30,583)	Training Set (n = 24,466)	Testing Set (n = 6117)
Age	64.4 ± 11.2	64.4 ± 11.2	64.3 ± 11.1
BMI	28.7 ± 6	28.7 ± 6	28.8 ± 5.9
Height (m)	1.7 ± 0.1	1.7 ± 0.1	1.7 ± 0.1
Weight (kg)	81.7 ± 20.6	81.6 ± 20.6	82.1 ± 20.6
Male Sex, n (%)	12,793 (41.8)	10,232 (41.8)	2561 (41.9)
Hip approach, n (%)			
Posterior	23,027 (75.3)	18,432 (75.3)	4595 (75.1)
Anterior	7556 (24.7)	6034 (24.7)	1522 (24.9)
Computer Navigation, n (%)	6233 (20.4)	4999 (20.4)	1234 (20.2)
Robotic arm assisted, n (%)	7322 (23.9)	5859 (24)	1463 (23.9)
First Race, n (%)			
White or Caucasian	26,306 (86)	21,046 (86)	5260 (86)
Black or African American	1971 (6.5)	1570 (6.4)	401 (6.6)
Asian	559 (1.8)	469 (1.9)	90 (1.5)
American Indian or Alaska native	72 (0.2)	60 (0.3)	12 (0.2)
Native Hawaiian or Other Pacific Islander	21(0.1)	14 (0.1)	7 (0.1)
Other	1654 (5.4)	1307 (5.3)	347 (5.6)

All continuous values are displayed as mean ± SD, categorical variables are displayed as count and percentage.

### 2.4 Model selection

Predictive performance and model interpretability were assessed to determine the best model. Performance was assessed with Root Mean Squared Error (RMSE) and Mean Absolute Error (MAE). 95 % confidence intervals (95 % CI) were calculated via bootstrapping. Furthermore, we assessed the number of correct predictions in the relevant ranges in intervals of ±2 mm.

We performed the analysis and model construction utilizing Python packages (v3.11, lightgbm, matplotlib, numpy, pandas, sklearn, InterpretML, RandomForestQuantileRegressor).

### 2.5 Feature importance

Feature weights were calculated from the models, which quantify the relative importance of each factor in predicting the outcome variable. Additionally, partial dependency plots (PDP) were generated for the weighted features of the EBM model to examine the functional relationship between the predictors and acetabular cup size at the population level. These plots facilitate the investigation of the directionality and shape of the associations, providing valuable insights for patients, surgeons, and other stakeholders involved in the clinical decision-making process.

## 3 Results

The study encompassed a total cohort of 30,583 patients. This patient population was then randomly split into two groups: a test set comprising 6117 patients and a training set of 24,466 patients. The distribution of variables in these groups is illustrated in Table 1.

### 3.1 Prediction model

Predicting the precise acetabular cup size, both models exhibited similar performance in terms of MAE, RMSE and Spearman correlation coefficient (Table 2). However, the QRF model demonstrated superior predictive ability in terms of correct prediction within ±2 mm intervals as well as limiting predictions to available cup sizes without intermediate values.

**Table 2:** Performance of Regression Models on test Set.

Model	MAE (95 %CI)	RMSE (95 %CI)	Spearman correlation coefficient (95 % CI)
EBM	1.73 (1.69, 1.77)	2.25 (2.18, 2.33)	0.8
QRF	1.69 (1.64, 1.73)	2.36 (2.29, 2.43)	0.78

CI = confidence interval; EBM = Explainable Boosting Machine; QRF = Quantile Regression Forest.

**Fig. 1:**
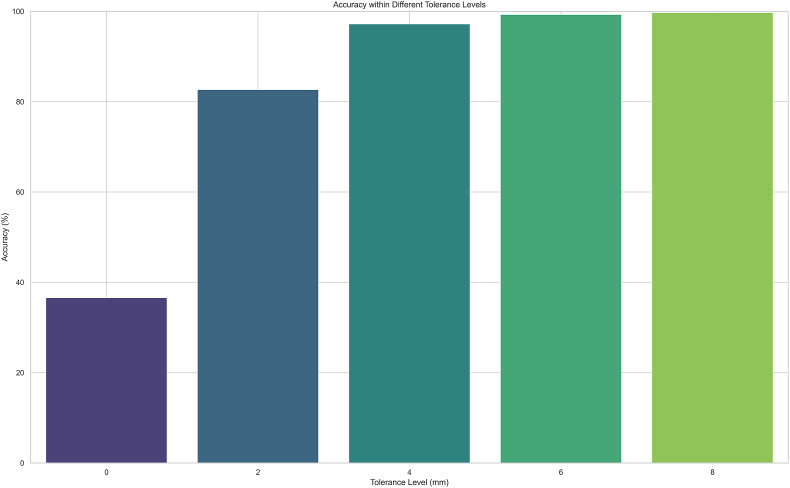
Bar plot displaying the accuracy of the QRF model in terms of correctly predicting acetabular cup size within ± _x mm intervals.

The QRF model predicted 82.85 % of cups correctly within ±2 mm and 97.27 % within ±4 mm (Fig. 1) while the EBM only achieved 65.41 % within ±2 mm and 93.56 % within ±4 mm (Table 3).

**Table 3:** Accuracy of Regression Models on test set.

Threshold	EBM	QRF
±0 mm	0 %	36.52 %
±2 mm	65.41 %	82.85 %
±4 mm	93.56 %	97.27 %
±6 mm	98.47 %	99.26 %
±8 mm	99.59 %	99.75 %

EBM = Explainable Boosting Machine; QRF = Quantile Regression Forest.

### 3.2 Feature importance

The features deemed most important in the QRF model were Sex, height (m), age, weight, surgical approach and BMI. Analysis of partial dependency plots of the EBM revealed that an increased probability of a larger cup size was associated with male sex, greater height, advanced age, higher weight, posterior approach and lower BMI. The relationships of continuous variables exhibited in the PDP can be considered linear. The QRF model structure does not allow for partial dependency plots.

### 3.3 Correlation of regression prediction

**Fig. 2:**
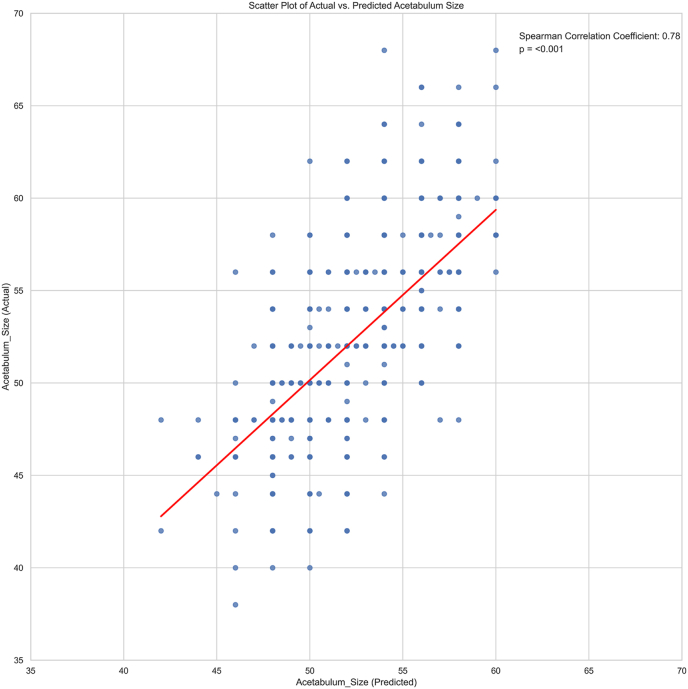
Scatterplot displaying the correlation of predicted and actual acetabular cup size.

Upon completing the regression analysis to predict acetabular cup size, the model outputs were correlated with the actual cup size using the Spearman correlation coefficient. The QRF did show an inferior correlation coefficient compared to the EBM model; however, the QRF was capable of natively predicting exact cup sizes without intermediate values (Fig. 2).

## 4 Discussion

In this study, we developed machine learning models to predict acetabular cup size before primary THA. We evaluated 2 machine learning (ML) algorithms. The discrepancies between the regression models were less recognizable when evaluating traditional metrics, however, were highlighted when applying the real-world use case of correctly predicting sizes in ± 2 mm intervals. We found that sex, height, age, weight, surgicalapproach and BMI were the most important predictors for acetabular cup size, all of which are readily available at the time of surgical consultation. To our knowledge, this is the largest single center study using Machine Learning to predict acetabular cup size.

Previous studies on implant size prediction explored similar variables to improve accuracy, however they demonstrated lower performance. Chen et al. utilized a dataset of 11,730 acetabular components implanted between 2005 and 2019 and applied a linear regression as well as a Bayesian model to predict both cup and stem size. Their model achieved an accuracy of 40.3 % within ± 2 mm and 73.5 % within ± 4 mm.^4^

Pourmoghaddam et al. built a model based on both demographic factors and radiographs, which achieved a very high accuracy of 93.4 % at ± 2 mm; however it has to be noted, this requires templating by the surgeon prior (and is the most heavily weighted variable of the model).^11^ As preoperative planning is typically carried out within a few days pre-operatively at our institution, the model would be more challenging to implement than that reported in this study.

If we assumed that orthopaedic companies provide 17 acetabular cup sizes per case for a weekly volume of 100 THAs in our institution the implementation of the current model would have resulted in the shipment of ~68.5 % fewer acetabular components. 1700 acetabular cups would be needed if we had all sizes for 100 THAs. If we assume 97.27 % accuracy at ±4 mm it would mean that for those 97.27 % of cases only 5 cup sizes would need to be shipped, for the remaining 2.73 % all cup sizes would have to be available. Therfore the average number of cups needed per case would be reduced from 17 to 5.33, extrapolated to 100 cases we can assume 1167 cups less required to be shipped.

This study has several limitations that should be considered when interpreting the results. As a single-center retrospective study focused solely on patients undergoing primary total joint arthroplasty, the generalizability of the findings may be limited to similar healthcare settings and patient populations. The retrospective design is subject to includes limited feature set of 9 variables may not capture all relevant factors influencing acetabular cup size. The lack of external validation on an independent dataset raises concerns about the true performance and generalizability of the models. While following the TRIPOD guidelines, the single-center design, lack of external validation, and potential biases should be considered when interpreting and applying the results.^6^ In addition, the model is based on the experice of a large orthopaedic institution, with surgeries performed by board-certified, sub-specialized surgeons. Unwanted intra-operative events can result in the need of implants that can be out of the predictive range. Such circumstances include but are not limited to excessive acetabular reaming and iatrogenic acetabular fracture, both of which can force the surgeon to utilize a larger component.

## 5 Conclusion

In conclusion, this study demonstrates that ML models can effectively predict acetabular cup size in patients undergoing primary THA. The provided both accurate predictions and interpretable insights into the most influential factors. The QRF offered superior capabilities in real world size prediction applications. Continued research is needed to further optimize the ML model and to evaluate its external validity.

## Guardian/patient's consent

A waiver of informed consent was granted in accordance with IRB guidelines, given that the study involves the use of de-identified data, ensuring patient confidentiality and privacy.

## Credit author statement

All listed authors have contributed substantially to this work: FCO, AIW and AGDV developed the idea for the present study. SL and YC were responsible for methodology and statistical analysis. FCO, AIW and AGDV performed primary manuscript preparation. Editing and final manuscript preparation was performed by DK, BC, AGDV, AIW and FCO. All authors read and approved the final manuscript.

## Ethical statement

This study has been reviewed and approved by the Institutional Review Board (IRB) under Expedited Review Category #5. The approval was granted on May 3, 2024, and will expire on May 2, 2027 (Study# 2024-0747). All necessary privacy and confidentiality measures, including the assignment of unique study numbers, will be strictly adhered to.

## Funding

This study was partially funded by the generous donation of the Peterson Foundation.

## Conflict of interest

SL is a paid consultant for CellSource. GCL receives royalties from Corin and Conformis and has stock or stock options in Corin and Parvizi Surgical Innovations. AGDV receives royalties from OrthoDeveloment, is a paid consultant for Johnson& Johnson, Link Orthopaedics, Naviswiss and OrthoDevelopment, furthermore he received research support from OrthoSensor.

## References

[1] Kurtz S., Ong K., Lau E., Mowat F., Halpern M. (2007 Apr). Projections of primary and revision hip and knee arthroplasty in the United States from 2005 to 2030. J Bone Joint Surg Am.

[2] Kurtz S.M., Lau E., Ong K., Zhao K., Kelly M., Bozic K.J. (2009). Future young patient demand for primary and revision joint replacement: national projections from 2010 to 2030. Clin Orthop Relat Res.

[3] Della Valle A.G., Padgett D.E., Salvati E.A. (2005 Nov). Preoperative planning for primary total hip arthroplasty. J Am Acad Orthop Surg.

[4] Chen J.B., Diane A., Lyman S., Chiu Y.F., Blevins J.L., Westrich G.H. (2022 Jun). Predicting implant size in total hip arthroplasty. Arthroplast Today.

[5] Pourmoghaddam A., Dettmer M., Freedhand A.M., Domingues B.C., Kreuzer S.W. (2015 Apr). A patient-specific predictive model increases preoperative templating accuracy in hip arthroplasty. J Arthroplast.

[6] Collins G.S., Reitsma J.B., Altman D.G., Moons K.G.M. (2015). Transparent reporting of a multivariable prediction model for individual Prognosis Or Diagnosis (TRIPOD): the TRIPOD statement. Br J Surg.

[7] Luo W., Phung D., Tran T. (2016). Guidelines for developing and reporting machine learning predictive models in biomedical research: a multidisciplinary view. J Med Internet Res.

[8] Nori H., Jenkins S., Koch P., Caruana R. (2019).

[9] Meinshausen N. (2006 06/01). Quantile regression forests. J Mach Learn Res.

[10] Johnson R.A. (2024). quantile-forest: a python package for quantile regression forests. J Open Source Softw.

[11] Arun N., Gaw N., Singh P. (2021). Assessing the trustworthiness of saliency maps for localizing abnormalities in medical imaging. Radiology: Artif Intell.

